# Supplementary material for: HSP90 inhibitors disrupt a transient HSP90-HSF1 interaction and identify a noncanonical model of HSP90-mediated HSF1 regulation
Source: Sci Rep. 2018 May 3;8:6976. doi: 10.1038/s41598-018-25404-w (PMC5934406; doi:10.1038/s41598-018-25404-w)
Supplement: Supplementary file 1 — Supplementary Information [file 41598_2018_25404_MOESM1_ESM.pdf]

Supplementary Information

HSP90 inhibitors disrupt a transient HSP90-HSF1 interaction and identify a noncanonical model of HSP90-mediated HSF1 regulation

Toshiki Kijima, Thomas L. Prince, Megan L. Tigue, Kendrick H. Yim, Harvey Schwartz, Kristin Beebe, Sunmin Lee, Marek A. Budzynski, Heinric Williams, Jane B. Trepel, Lea Sistonen, Stuart Calderwood and Len Neckers

Kijima et al. Supplemental Information

Methods

Plasmids: The HSF1 HR-A/B mutants145A_4 (Q145A, M147A, K148A, K150A), 151A_5 (Q151A, E152A, C153A, M154A, D155A) and 161A_4 (M161A, K162A, H163A, Q164A) were made using Quickchange method. The pcDNA-FLAG-dPRD HSF1 constructs were made by inserting the dPRD HSF1 coding region from pcDNA3.1/Myc-His-HSF1 dPRD (REF) into pcDNA3-FLAG by Gibson assembly. The plasmids for luciferase reporter assay, pGL3-Hsp70-LUC, pGL3-CMV-LUC, and pCMV-βGal were made or obtained earlier (1).

Chromatin immunoprecipitation and PCR: Chromatin immunoprecipitation experiments were carried out according the protocol described by Nelson et al (2). Real time PCR was performed to quantify HSF1 binding to the HSPA1A (HSP70) promoter. HSPA1A primer sequences (MWG-Operon): 5'CTGTCAATTAGGCGCTGAAG3', 5'TCTTCTGGGATTCACTGGAG3'. Cells were harvested over the described time course. Endogenous *HSP70* mRNA expression was detected by RT-qPCR using the ABI 7500 Real Time Detection System (Life Technologies) as described previously (3). In brief, total RNA was isolated from cultured cells using the RNeasy RNA Isolation Kit (Qiagen) and reverse transcribed using the Maxima First Strand cDNA Synthesis Kit (Thermo Scientific). RT-qPCR experiments were performed in triplicate. All primer sequences are as follows:

rRNA: forward 5'- AGTCCCTGCCCTTTGTACACA-3', reverse 5' CGATCCGAGGGCCTCACTA-3'; HSP70: forward 5'- GCCCTGATCAAGCGCAACT-3', reverse 5'-TTGTCGGAGTAGGTGGTGAAGA-3'.

Luciferase reporter assays: HEK293 cells were co-transfected with pGL3-CMV-LUC, pCMV-βGal, and a series of deletion constructs of pcDNA-FLAG-HSF1 by using X-tremeGENE 9 transfection reagent. With or without 1-hour pretreatment with 10 μM 17-AAG, cells were heat shocked at 42°C for 30 minutes, then incubated at 37°C for 4 hours. An additional group was exposed to 17AAG without heat shock. Cells were lysed with reporter lysis buffer (25 mM Bicine, 0.05% Tween20, 0.05% Tween80). Luciferase activity was determined with luciferase assay buffer (25 mM Glycine, 15 mM KPO_4, 15 mM $MgSO_4$, 4 mM EGTA, 2 mM ATP, 1 mM DTT, 1.7 mM potassium luciferin), and normalized to the βGal activity of each well. All measurements were performed at least in triplicate. Data are shown as mean ± standard deviation. All statistical analysis was done in Excel.

LUMIER analysis: FLAG-tagged HSF1 constructs were transfected into HEK293 cells growing in 96-well plates and stably expressing WT NanoLuc(NL)-HSF1. After 18 hours, cells were harvested by washing with cold PBS, lysing with TGNET complete buffer and pelleted in a table top centrifuge at 3,000 rpm at 4ºC. Lysates were transferred to an anti-FLAG antibody-coated plate and incubated at 4ºC with gentle shaking for 2 hrs. The plates were then washed 3 times with TGNET buffer and Nano-Glo reagent was added. Luciferase activity was read and quantified using a luminometer plate reader. Each construct was assayed 8 times. Standard deviations are represented by error bars.

Supplementary Figure Legends

Supplementary Figure 1

A. Expression levels of endogenous HSF1 and transfected FLAG-HSF1 WT in HEK293 cells. HEK293 cells were transfected with pcDNA or FLAG-HSF1 and analyzed by HSF1 WB. Actin WB was used as a loading control.

B. Interaction between HSP90α E47A and endogenous HSF1. HEK293 cells were transfected with either pcDNA or FLAG-HSP90α E47A and analyzed by HSF1 IP – FLAG (HSP90) WB.

C. Disruption of HSF1 interaction with “closed” HSP90α E47A/HSP90β E42A mutants by N-terminal but not C-terminal HSP90 inhibitors. HEK293 cells were transfected with HA-HSF1 and FLAG-HSP90α constructs (upper panel) or HSP90β constructs (lower panel), treated with 17AAG (N-terminal inhibitor) or KU32 (C-terminal inhibitor) and analyzed by anti-FLAG (HSP90) IP – HSF1 WB.

Full blots used to create cropped figure panels are shown in Supplementary Figure 6.

Supplementary Figure 2

A. Interaction of FLAG-HSF1 C-terminal truncation constructs with endogenous (wild type) HSP90, HA-HSP90α E47A, and endogenous (wild type) HSP70. HEK293 cells were transfected with FLAG-HSF1 and either pcDNA or HA-HSP90α E47A and then analyzed by anti-FLAG IP followed by HSP90, HA, or HSP70 WB.

B. Interaction of HSF1 N-terminal truncation and internal deletion constructs with endogenous HSP90, HA- HSP90α E47A, HA- HSP90β E42A, and endogenous HSP70. HEK293 cells were transfected and analyzed as in panel A.

C. Deletion of portions of the HR-A/B domain prevents HSF1 dimerization. HEK293 cells were transfected with WT HA-HSF1 and FLAG-HSF1 WT, alanine or internal deletion mutants then analyzed by anti-FLAG IP - HA WB.

D. Alteration of the HSF1 HR-A/B domain increases HSP90 interaction. HEK293 cells were transfected with HA-HSP90α E47A and FLAG-HSF1 WT, alanine or internal deletion mutants, then analyzed by anti-FLAG IP - HA WB. Unlike HSF1 dimerization, association with HSP90α E47A was enhanced by both HSF1 alanine mutation or internal deletion when compared to WT.

E. Key HSF1 C-terminal truncation mutants show increased binding in HSF1 knockdown cells. Endogenous HSF1 expression was knocked down in HEK293 cells by stable expression of HSF1-directed shRNA and these cells were used to repeat FLAG (HSF1) IP- HA (HSP90α E47A) WB analysis from Figure 2C. A representative WB is shown (left panel). This experiment was performed in triplicate and all three experiments are shown in Supplementary Figure 6. The right panel shows the efficiency of stable knock down of endogenous HSF1 in these HEK293 cells.

Full blots used to create cropped figure panels are shown in Supplementary Figure 6.

Supplementary Figure 3

A. *hsp70* promoter-driven luciferase activity in HEK293 cells subjected to heat shock alone, 17AAG alone, or heat shock after pre-exposure to 17AAG, was assessed as described in Supplemental Methods (4 hours after recovery from heat shock, equivalent to 5.5 hours after addition of 17AAG).

B. P-values indicate statistically significant differences in *hsp70* promoter-reporter activity of mutant HSF1 transfected cells as compared to cells transfected with WT HSF1 or pcDNA control in heat shock alone and 17AAG + heat shock conditions. Cells were analyzed as described in Figure 3A and C. A one-way ANOVA utilizing multiple comparisons (Sidak test) was performed to determine significant Differences as shown.

C. FLAG-HSF1 constructs containing the HR-A/B domain and at least part of the RD dimerize with WT HSF1. HEK293 cells were transfected with WT HA-HSF1 and FLAG-HSF1 deletion constructs, then analyzed by anti-FLAG IP and HA WB.

D. HSF1 trimerization requires HR-A/B domain. Deletion of HR-A/B (F-N195-C383, F-N262) abolishes HSF1 trimerization. Deletion of the DBD (F-N120) and TAD (F-N120-C383, F-N120-C325, F-N120-C287) increases HSF1 trimerization and allows for dominant negative repression of HSF1 transcription activity. Relative interaction data were obtained by LUMIER analysis (see Supplemental Methods).

E. Phosphorylation state of the RD does not significantly affect HSF1 trimerization. Both full length F-HSF1 WT and F-dPRD HSF1 bind NL-HSF1 to a similar degree. Relative interaction data were obtained by LUMIER analysis (see Supplemental Methods).

Supplementary Figure 4

Treatment with 17AAG causes phosphorylation outside of the RD domain of HSF1. HEK293 cells were transfected with either F-HSF1 WT or F-HSF1 dPRD mutant. Indicated samples were then pretreated 24 hours later with 10 μM 17AAG, an N-terminal HSP90 inhibitor, for 4 hours. Cells were lysed and the lysates subjected to FLAG IP, followed by treatment of the IP with lambda phosphatase, and finally HSF1 WB analysis.

Full blots used to create cropped figure panels are shown in Supplementary Figure 6.

Supplementary Figure 5

A. Schema of the binding profiles of HSF1 constructs to endogenous HSP90, HSP90α E47A, HSP90β E42A and HSP70, and the ability of each truncation and deletion construct to trimerize with WT HSF1.

B. Schema of the approximate locations on HSF1 where HSP90 and HSP70 interact, based on data reported in this study.

Supplementary Figure 6

A compilation of full blots used to produce main and supplemental figures as designated.

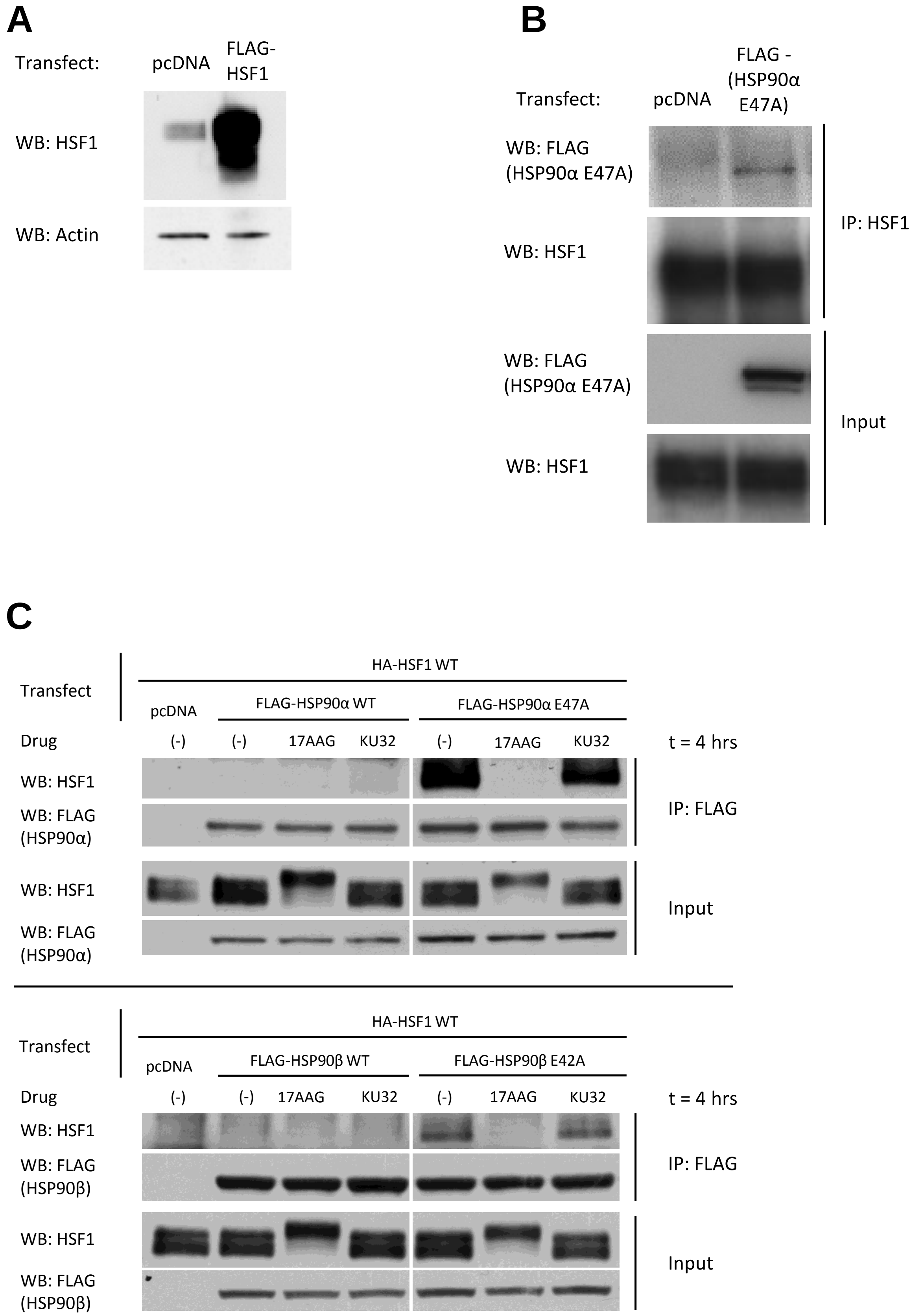
A
Transfect:
pcDNA
FLAG-HSF1
WB: HSF1
WB: Actin
B
Transfect:
pcDNA
FLAG - (HSP90α E47A)
WB: FLAG (HSP90α E47A)
WB: HSF1
IP: HSF1
WB: FLAG (HSP90α E47A)
WB: HSF1
Input
C
Transfect
HA-HSF1 WT
pcDNA
FLAG-HSP90α WT
FLAG-HSP90α E47A
Drug
(-)
(-)
17AAG
KU32
(-)
17AAG
KU32
t = 4 hrs
WB: HSF1
WB: FLAG (HSP90α)
IP: FLAG
WB: HSF1
WB: FLAG (HSP90α)
Input
Transfect
HA-HSF1 WT
pcDNA
FLAG-HSP90β WT
FLAG-HSP90β E42A
Drug
(-)
(-)
17AAG
KU32
(-)
17AAG
KU32
t = 4 hrs
WB: HSF1
WB: FLAG (HSP90β)
IP: FLAG
WB: HSF1
WB: FLAG (HSP90β)
Input

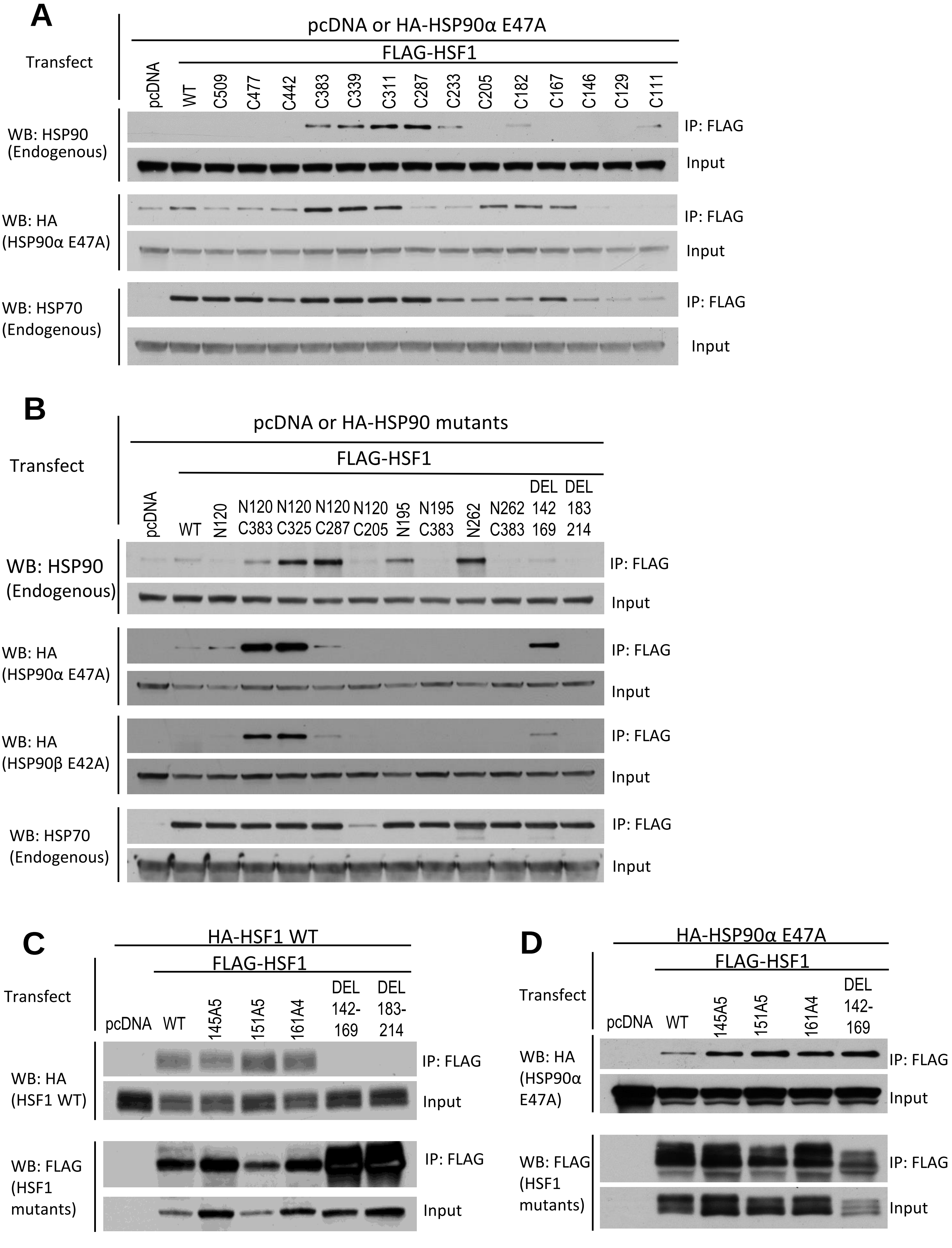
A
Transfect
pcDNA or HA-HSP90α E47A
FLAG-HSF1
pcDNA
WT
C509
C477
C442
C383
C339
C311
C287
C233
C205
C182
C167
C146
C129
C111
WB: HSP90 (Endogenous)
WB: HA (HSP90α E47A)
WB: HSP70 (Endogenous)
IP: FLAG
Input
B
Transfect
pcDNA or HA-HSP90 mutants
FLAG-HSF1
pcDNA
WT
N120
N120 C383
N120 C325
N120 C287
N120 C205
N195
N195 C383
N262
N262 C383
DEL 142 169
DEL 183 214
WB: HSP90 (Endogenous)
WB: HA (HSP90α E47A)
WB: HA (HSP90β E42A)
WB: HSP70 (Endogenous)
IP: FLAG
Input
C
Transfect
HA-HSF1 WT
FLAG-HSF1
pcDNA
WT
145A5
151A5
161A4
DEL 142-169
DEL 183-214
WB: HA (HSF1 WT)
WB: FLAG (HSF1 mutants)
IP: FLAG
Input
D
Transfect
HA-HSP90α E47A
FLAG-HSF1
pcDNA
WT
145A5
151A5
161A4
DEL 142-169
WB: HA (HSP90α E47A)
WB: FLAG (HSF1 mutants)
IP: FLAG
Input

E

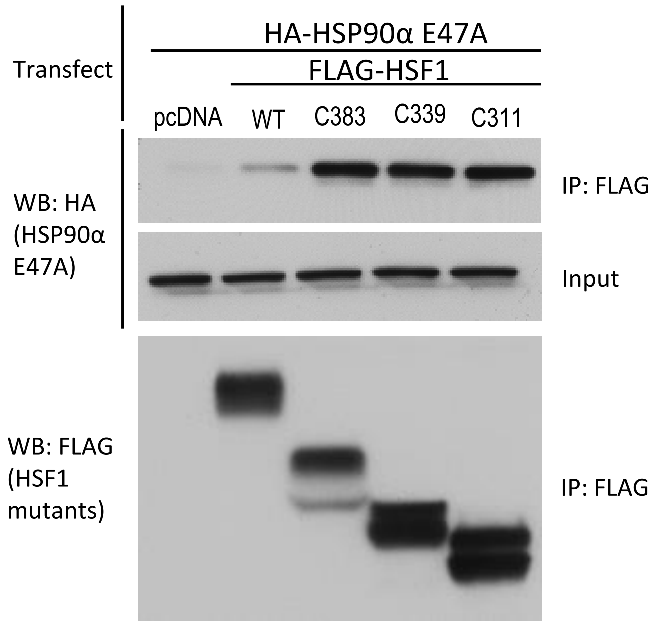
Transfect
HA-HSP90α E47A
FLAG-HSF1
pcDNA
WT
C383
C339
C311
WB: HA (HSP90α E47A)
IP: FLAG
Input
WB: FLAG (HSF1 mutants)
IP: FLAG

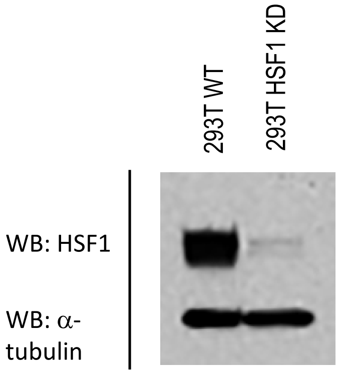
293T WT
293T HSF1 KD
WB: HSF1
WB: α-tubulin

A

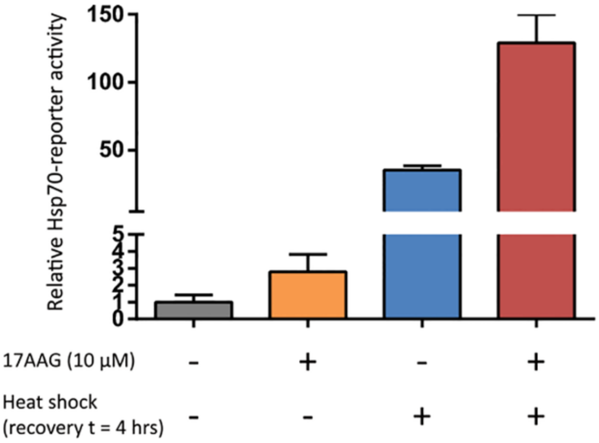

B

Heat Shock Figure 3A		
Construct	p-values	
pcDNA	pcDNA vs	WT vs
WT	<0.0001	
C509	<0.0001	0.001
C477	>0.9999	<0.0001
C442	<0.0001	<0.0001
C383	<0.0001	<0.0001
C339	<0.0001	<0.0001
C311	<0.0001	<0.0001
C287	<0.0001	<0.0001
C233	<0.0001	<0.0001
C205	<0.0001	<0.0001
C182	>0.9999	<0.0001
C167	<0.0001	<0.0001
C146	0.0018	<0.0001
C129	0.0139	<0.0001
C111	0.0004	<0.0001

17AAG + HS Figure 3A		
Construct	p-values	
pcDNA	pcDNA vs	WT vs
WT	<0.0001	
C509	0.0001	0.3423
C477	0.5549	<0.0001
C442	<0.0001	<0.0001
C383	<0.0001	<0.0001
C339	<0.0001	<0.0001
C311	<0.0001	<0.0001
C287	<0.0001	<0.0001
C233	<0.0001	<0.0001
C205	<0.0001	<0.0001
C182	>0.9999	<0.0001
C167	0.0111	0.0065
C146	0.8813	<0.0001
C129	>0.9999	<0.0001
C111	>0.9999	<0.0001

Heat shock Figure 3C		
Construct	p-values	
pcDNA	pcDNA vs	WT vs
WT	0.9637	
N120	0.3902	0.0007
N120-C383	0.3758	0.0007
N120-C325	0.3895	0.0007
N120-C287	0.8825	0.0045
N195	<0.0001	<0.0001
N195-C383	<0.0001	<0.0001
N195-C325	<0.0001	<0.0001
N262	<0.0001	0.0009
N262-C383	<0.0001	0.0003
N262-C325	<0.0001	0.0052
Del142-69	<0.0001	<0.0001
Del183-214	<0.0001	<0.0001

17AAG + HS Figure 3C		
Construct	p-values	
pcDNA	pcDNA vs	WT vs
WT	0.9157	
N120	>0.9999	0.4
N120-C383	>0.9999	0.3966
N120-C325	>0.9999	0.3742
N120-C287	>0.9999	0.4008
N195	0.9408	>0.9999
N195-C383	0.6124	>0.9999
N195-C325	0.728	>0.9999
N262	0.9851	>0.9999
N262-C383	0.9939	>0.9999
N262-C325	0.9966	>0.9999
Del142-69	0.0738	>0.9999
Del183-214	0.0074	0.9846

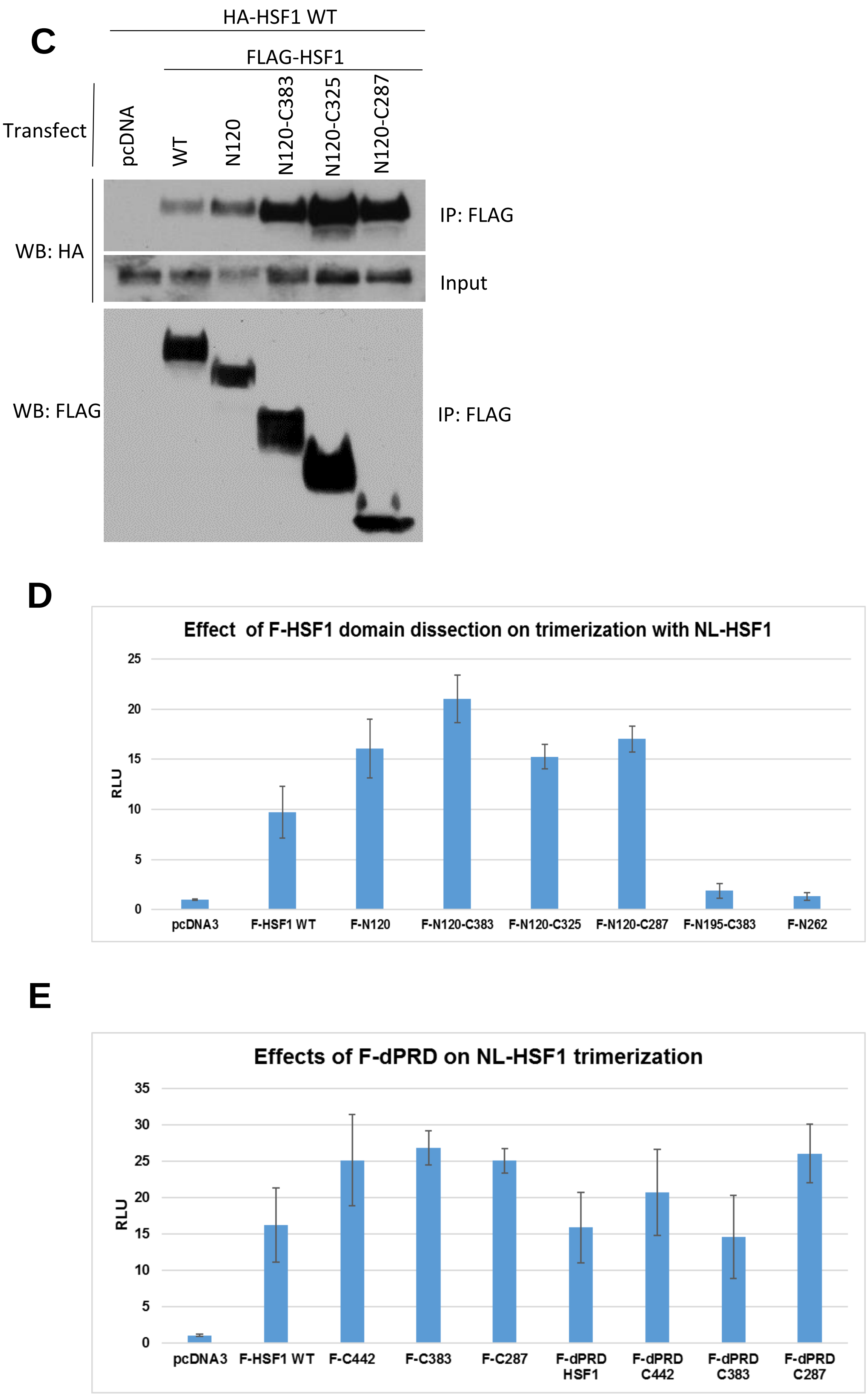
C
HA-HSF1 WT
FLAG-HSF1
Transfect
pcDNA
WT
N120
N120-C383
N120-C325
N120-C287
WB: HA
IP: FLAG
Input
WB: FLAG
IP: FLAG
D
Effect of F-HSF1 domain dissection on trimerization with NL-HSF1
RLU
25
20
15
10
5
0
pcDNA3
F-HSF1 WT
F-N120
F-N120-C383
F-N120-C325
F-N120-C287
F-N195-C383
F-N262
E
Effects of F-dPRD on NL-HSF1 trimerization
RLU
35
30
25
20
15
10
5
0
pcDNA3
F-HSF1 WT
F-C442
F-C383
F-C287
F-dPRD HSF1
F-dPRD C442
F-dPRD C383
F-dPRD C287

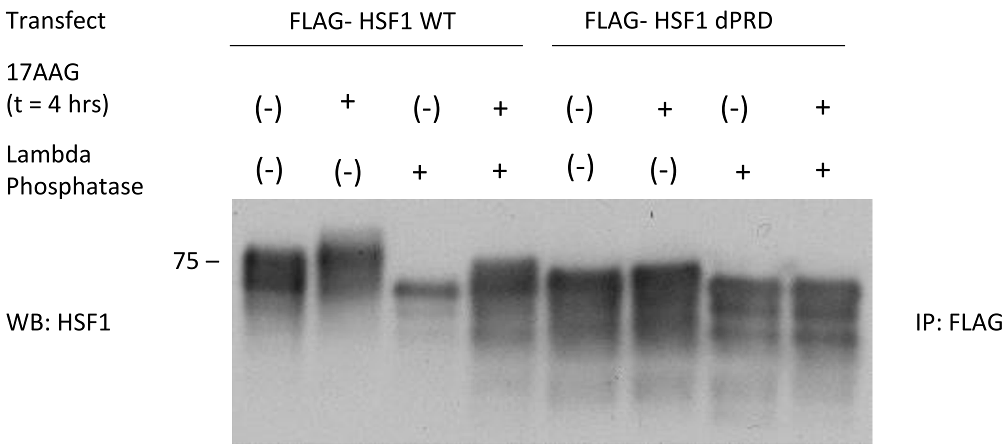
Transfect
FLAG- HSF1 WT
FLAG- HSF1 dPRD
17AAG
(t = 4 hrs)
(-) + (-) + (-) + (-) +
Lambda
Phosphatase
(-) (-) + + (-) (-) + +
75 –
WB: HSF1
IP: FLAG

A

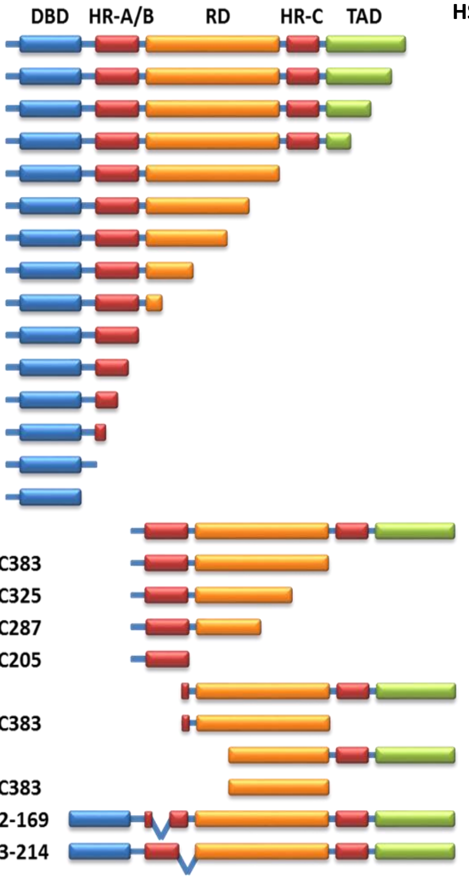

	Endogenous HSP90	HSP90α E47A	HSP90β E42A	Endogenous HSP70	Trimerization
WT	-	+	+/-	++	+
C509	-	+/-	ND	++	+
C477	-	+/-	ND	++	+
C442	-	-	ND	++	+
C383	+	++	ND	++	+
C339	+	++	ND	++	+
C311	+	++	ND	++	+
C287	+	+/-	ND	++	+
C233	+/-	+/-	ND	+	+
C205	-	+	ND	+	+
C182	+/-	+	ND	+	-
C167	-	+	ND	+	-
C146	-	+/-	ND	+/-	-
C129	-	+/-	ND	+/-	-
C111	-	-	ND	+/-	ND
N120	-	-	+/-	+	+
N120-C383	+	+/-	++	+	+
N120-C325	++	++	++	+	+
N120-C287	++	+	+/-	+	+
N120-C205	-	-	-	-	ND
N195	+	-	-	+	-
N195-C383	-	-	-	+	-
N262	++	-	-	+	-
N262-C383	-	-	-	+	ND
DEL142-169	-	++	+	+	-
DEL183-214	-	-	-	+	-

- No interaction
+/- Weak interaction
+ Mild interaction
++ Strong interaction
ND no data

B

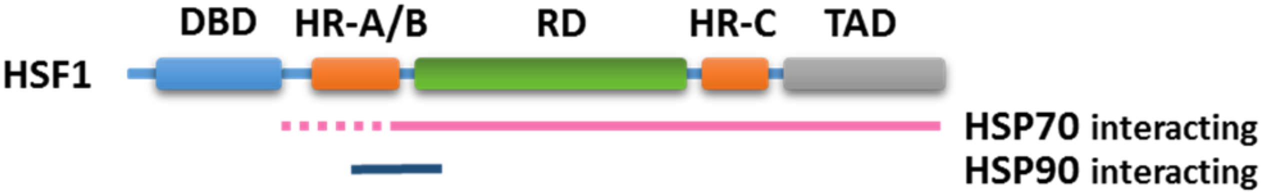

Figure 1

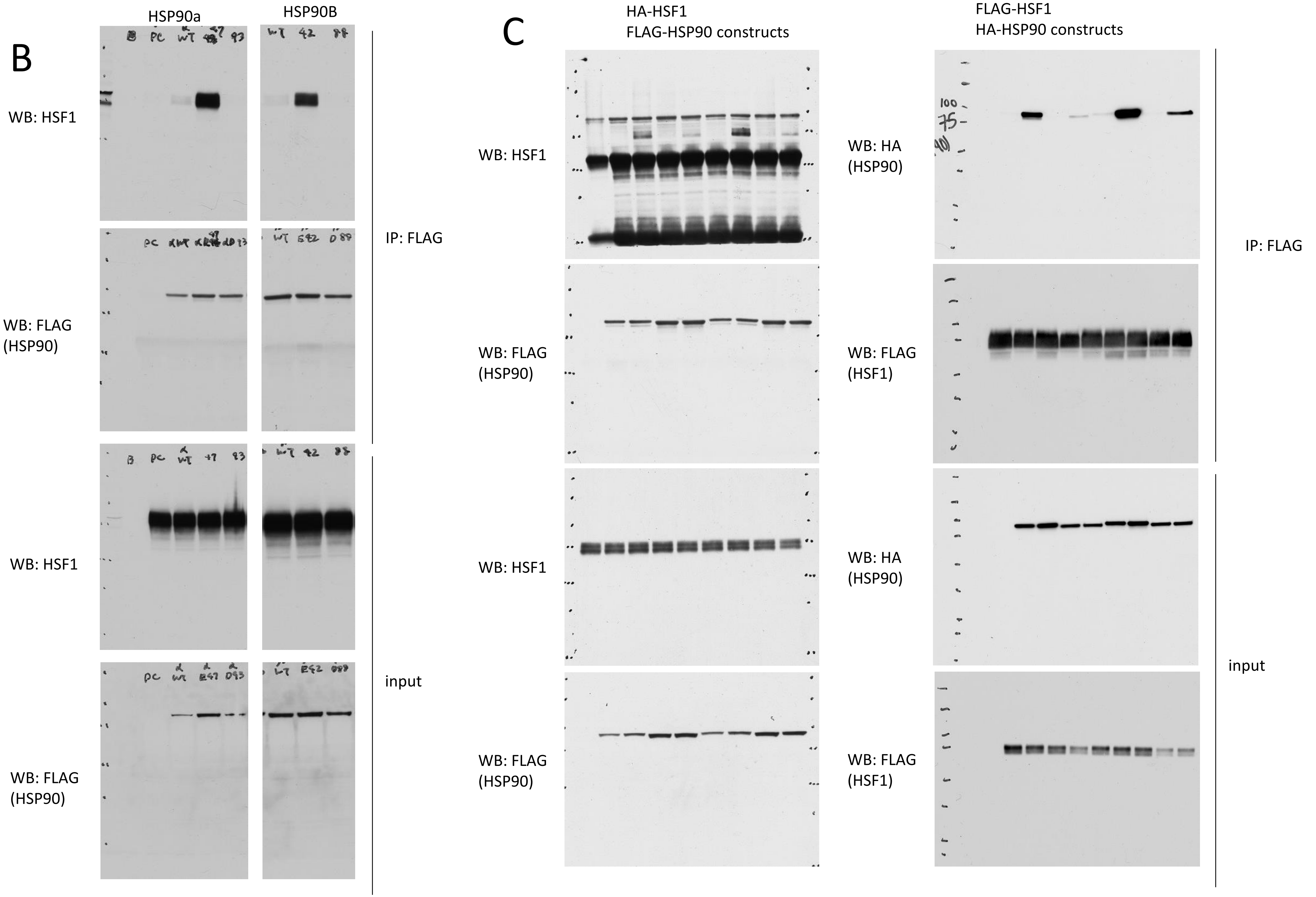
B
HSP90a
HSP90B
WB: HSF1
WB: FLAG
(HSP90)
IP: FLAG
WB: HSF1
WB: FLAG
(HSP90)
input
C
HA-HSF1
FLAG-HSP90 constructs
FLAG-HSF1
HA-HSP90 constructs
WB: HSF1
WB: FLAG
(HSP90)
WB: HSF1
WB: FLAG
(HSP90)
WB: HA
(HSP90)
WB: FLAG
(HSF1)
WB: HA
(HSP90)
WB: FLAG
(HSF1)
IP: FLAG
input

Figure 1 cont.

D

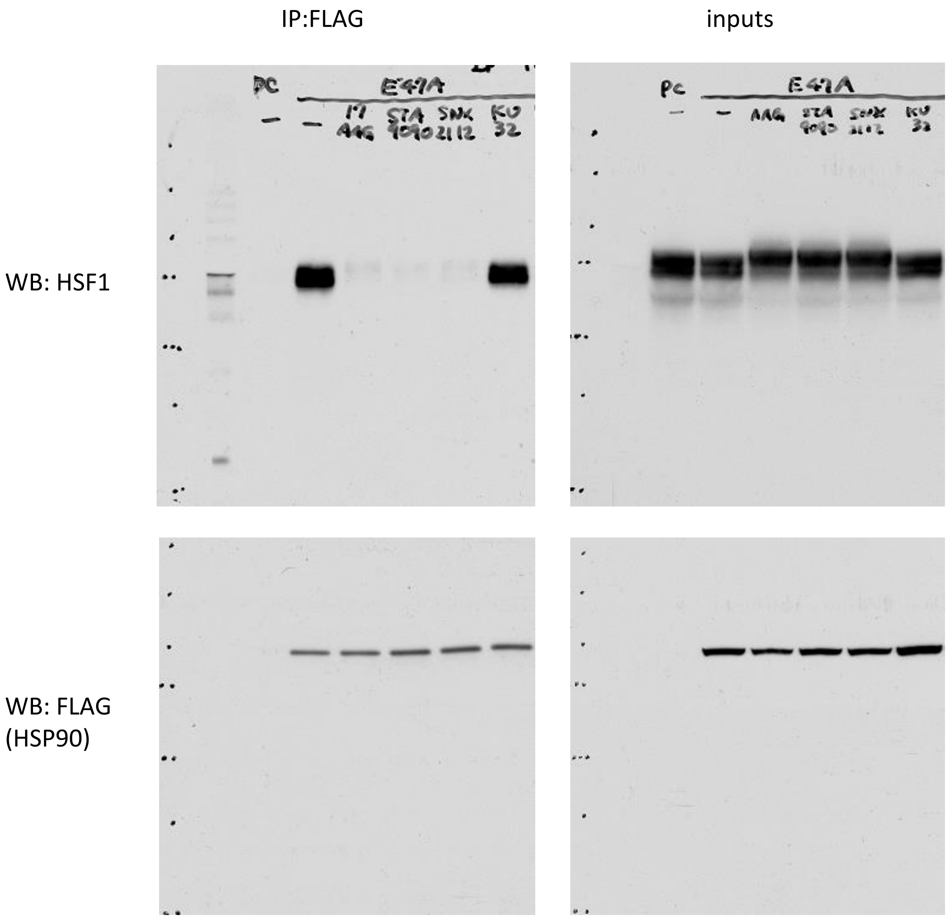

Figure 2

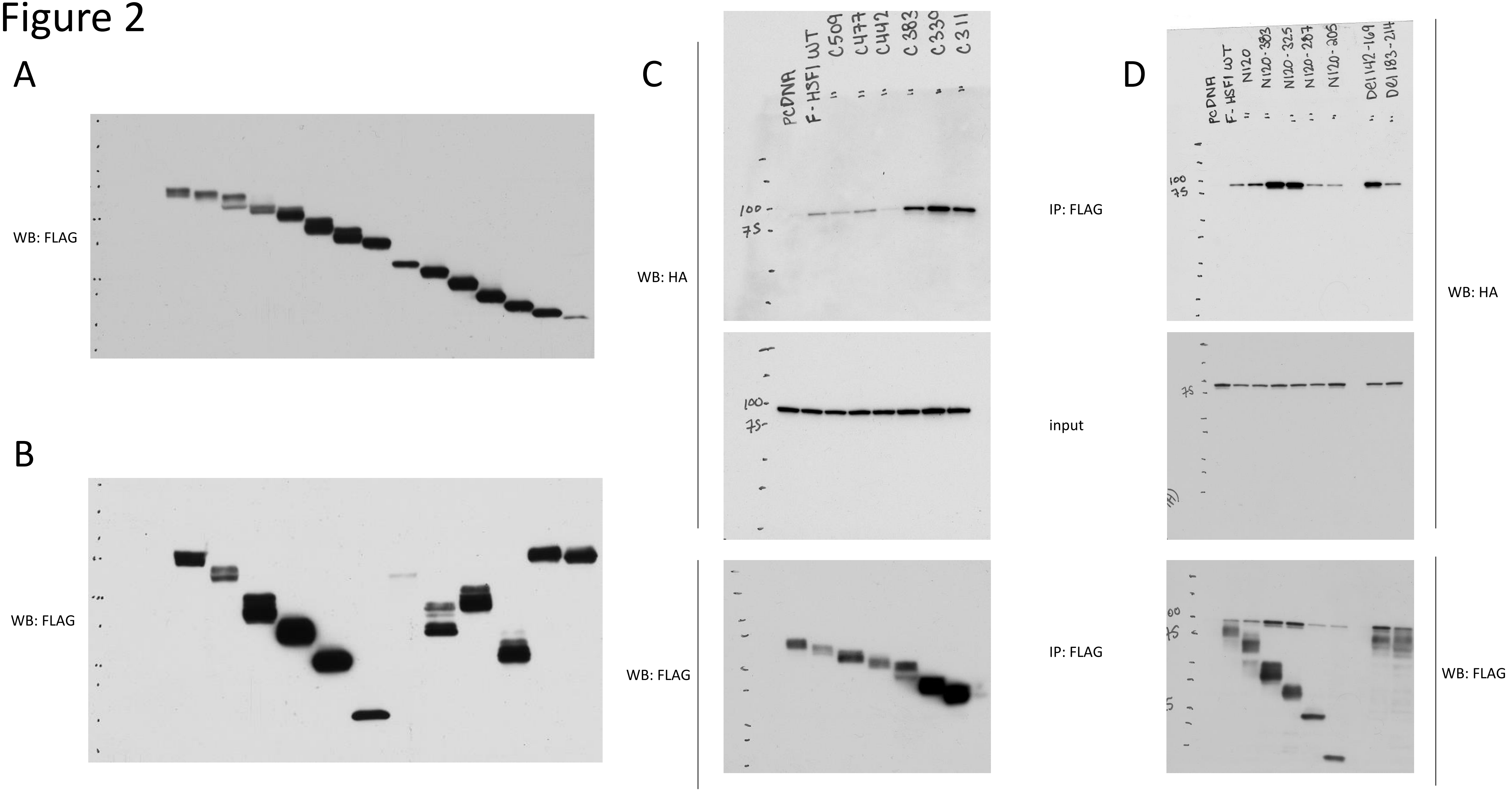
A
WB: FLAG
B
WB: FLAG
C
WB: HA
WB: FLAG
pcDNA
F-HSF1 WT
C509
C477
C442
C383
C330
C311
100
75
IP: FLAG
input
IP: FLAG
D
pcDNA
F-HSF1 WT
N120
N120-383
N120-325
N120-287
N120-205
Del142-169
Del183-214
WB: HA
WB: FLAG

2C

Replicates 2 and 3

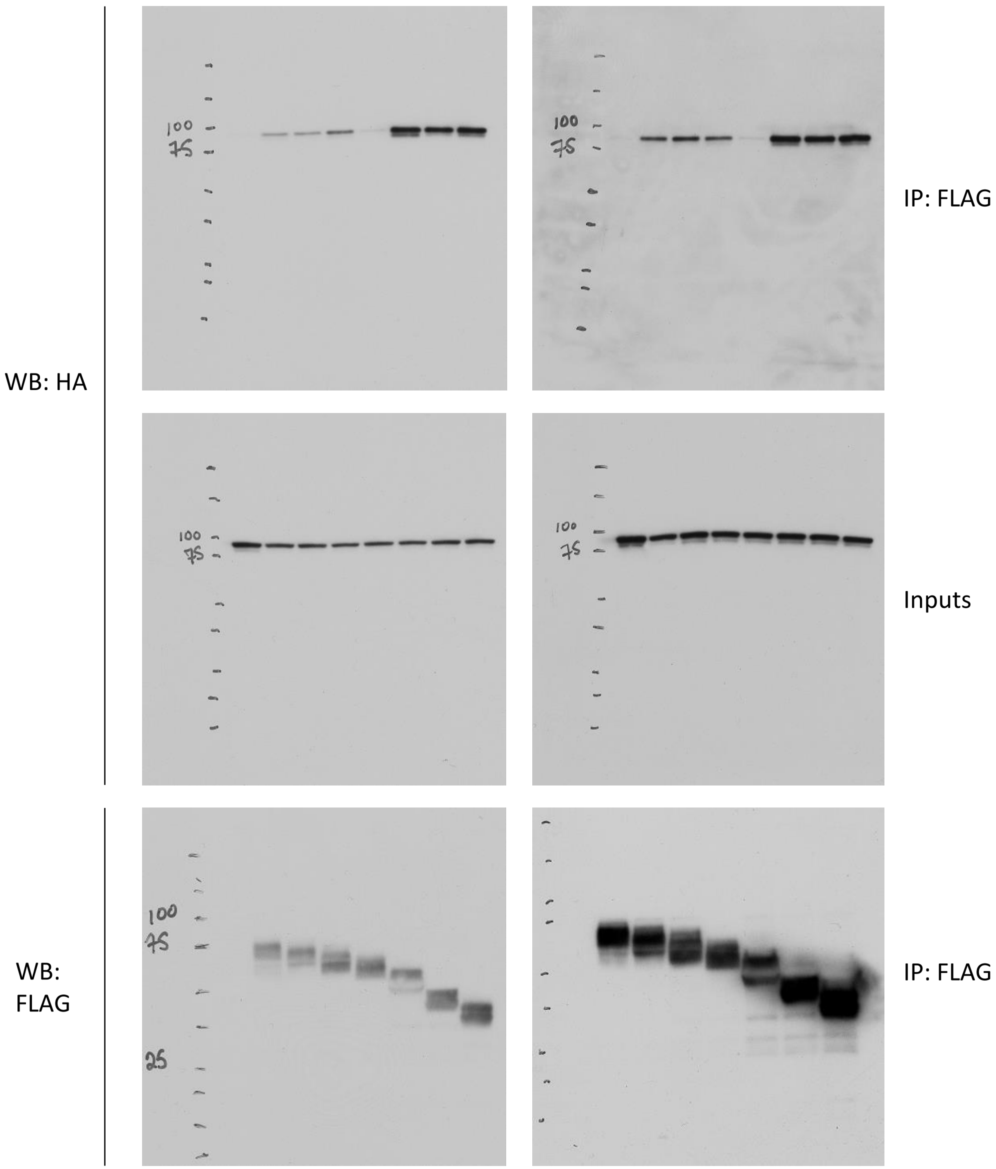

2D

Replicates 2 and 3

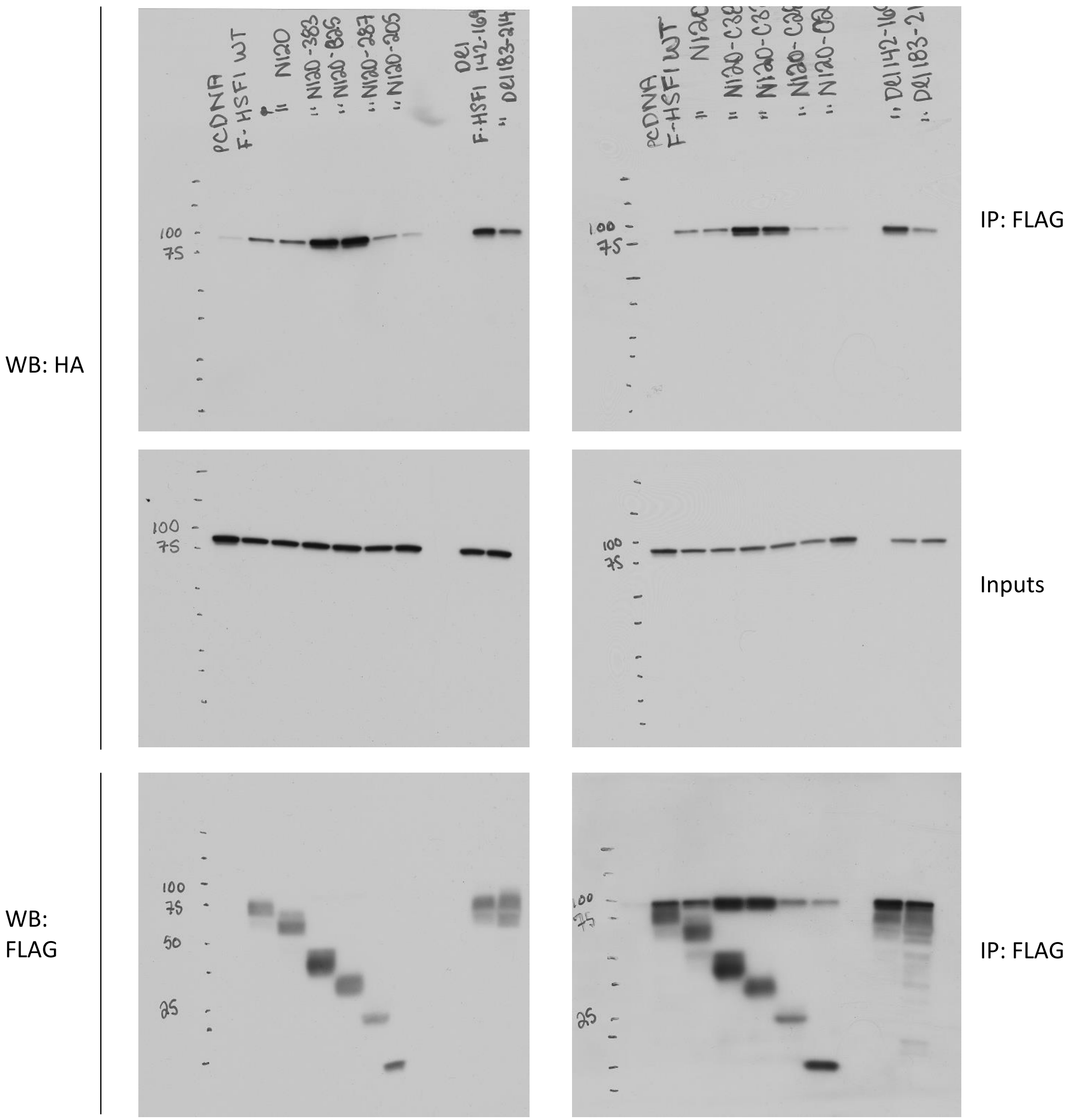

Figure 3

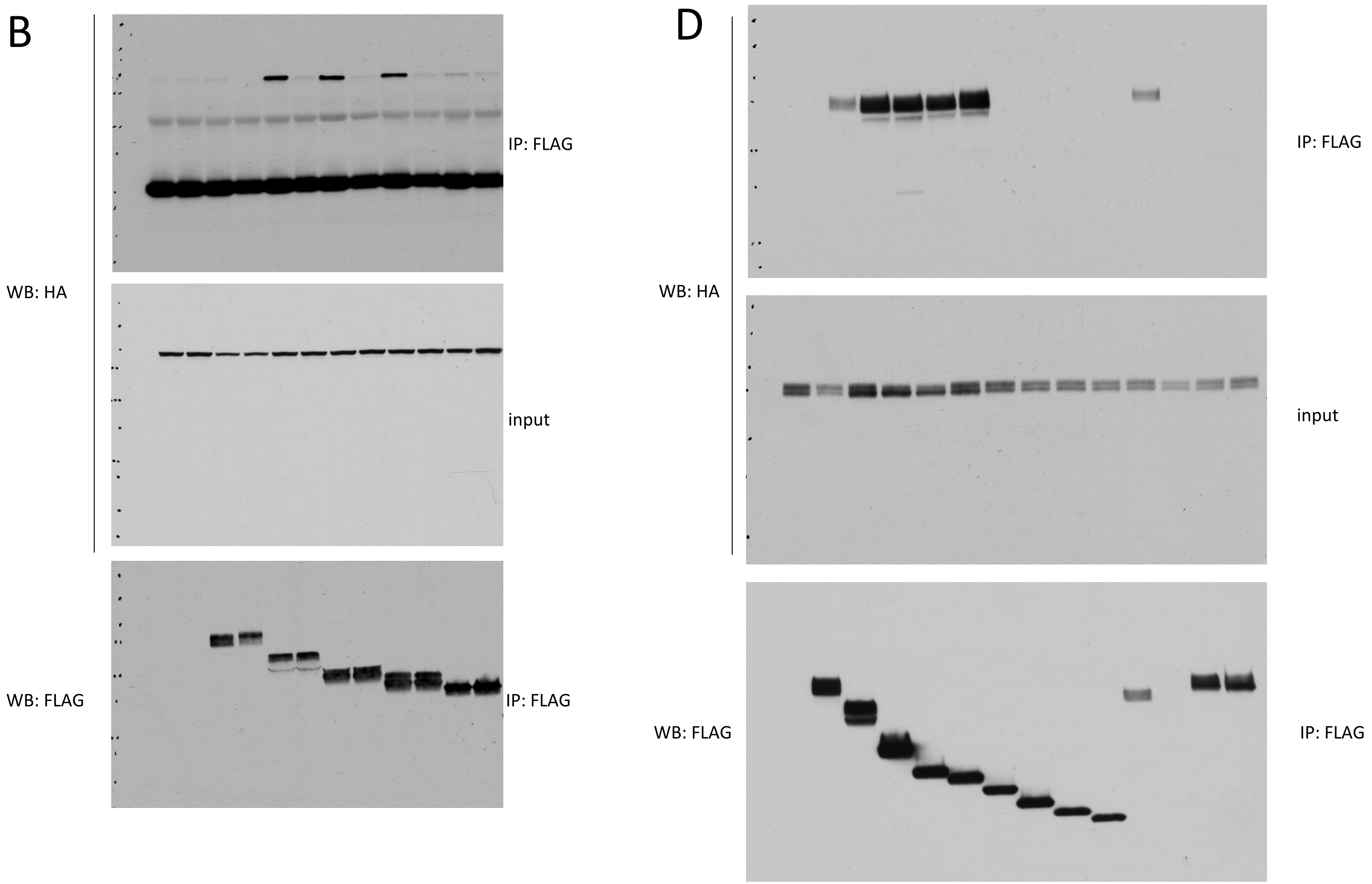
B
IP: FLAG
WB: HA
input
WB: FLAG
IP: FLAG
D
IP: FLAG
WB: HA
input
WB: FLAG
IP: FLAG

Figure 4

C

WB: HSF1

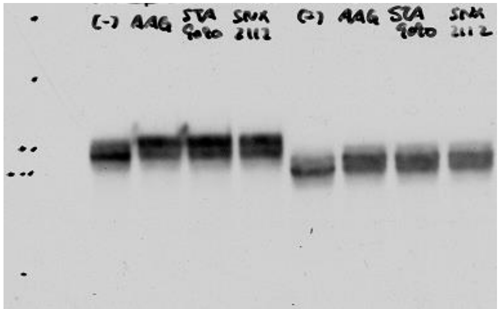

Figure 5

A

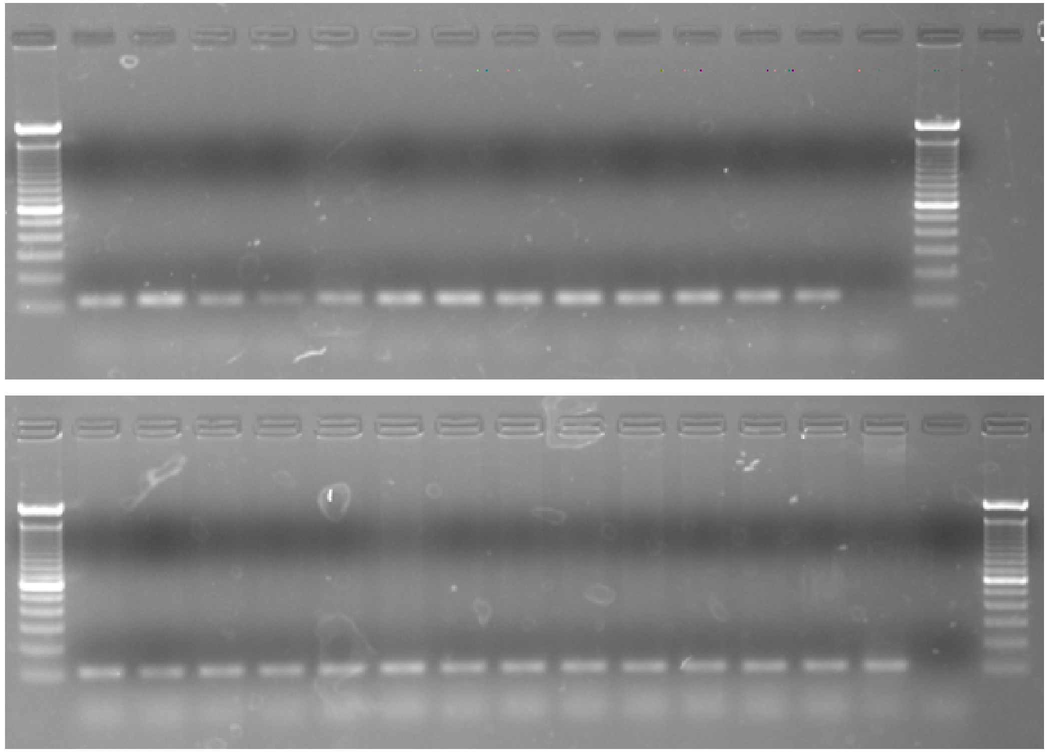

Supplemental Figure 1

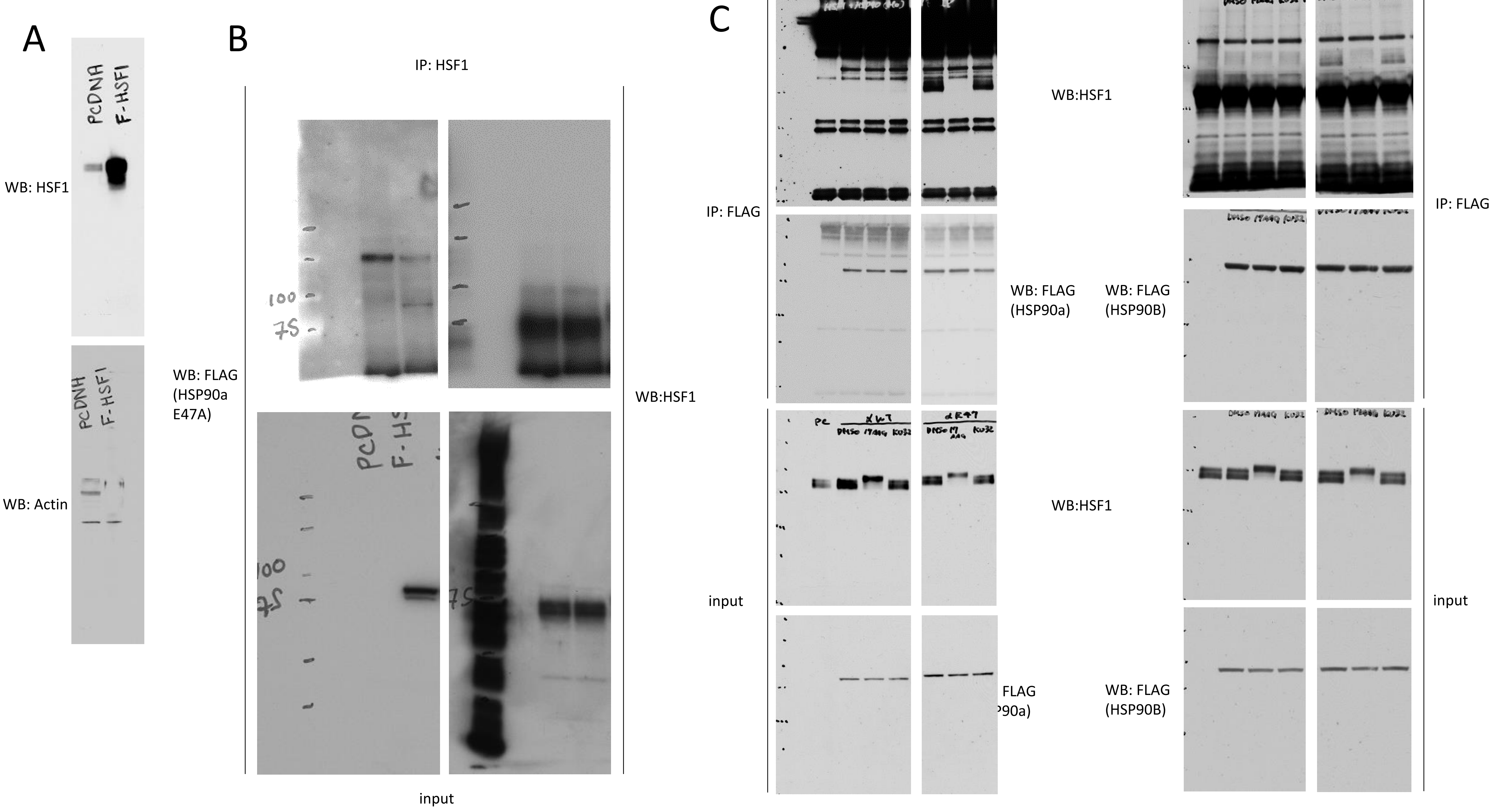

Supplemental Figure 2

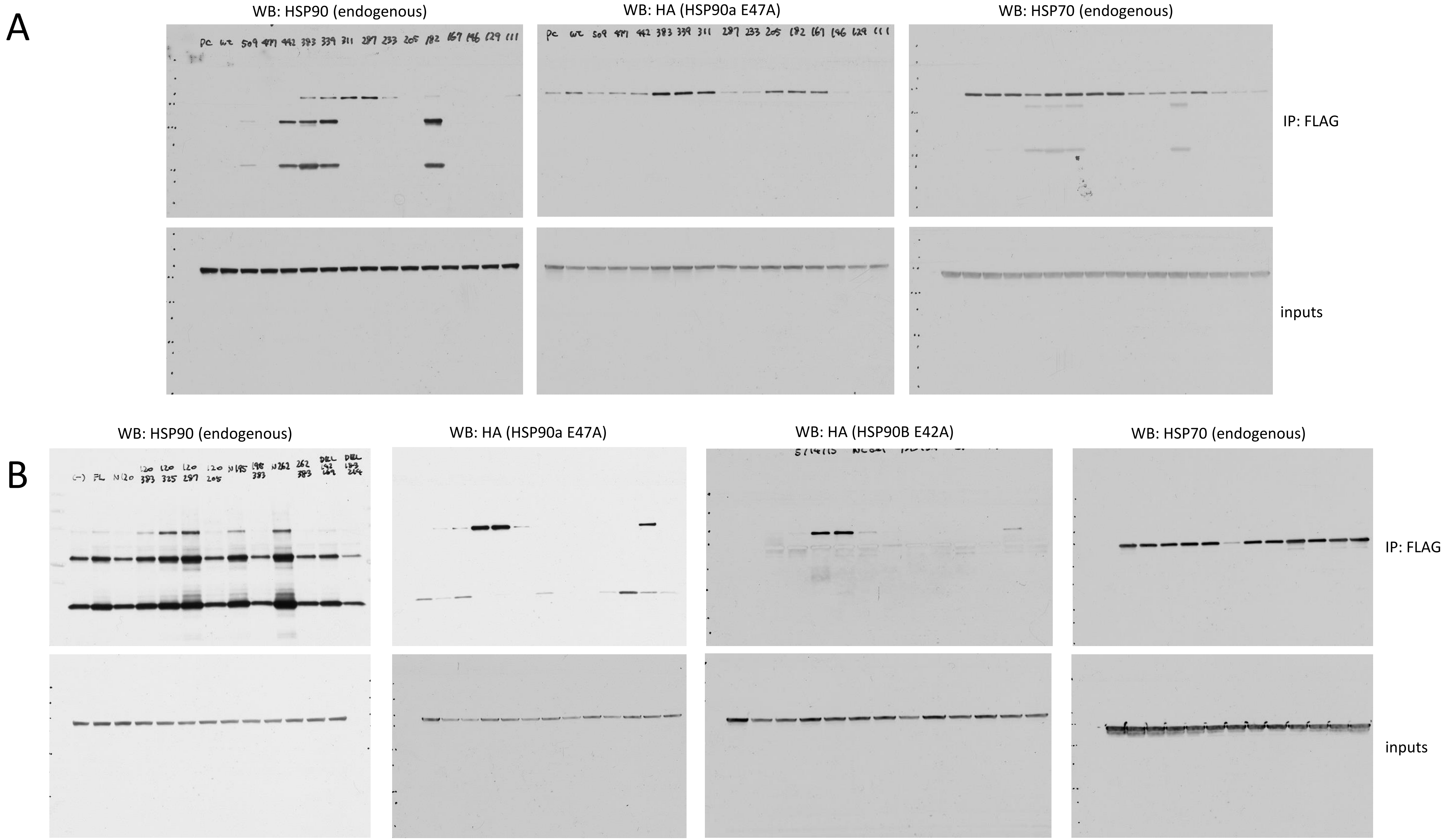

Supplemental Figure 2 cont.

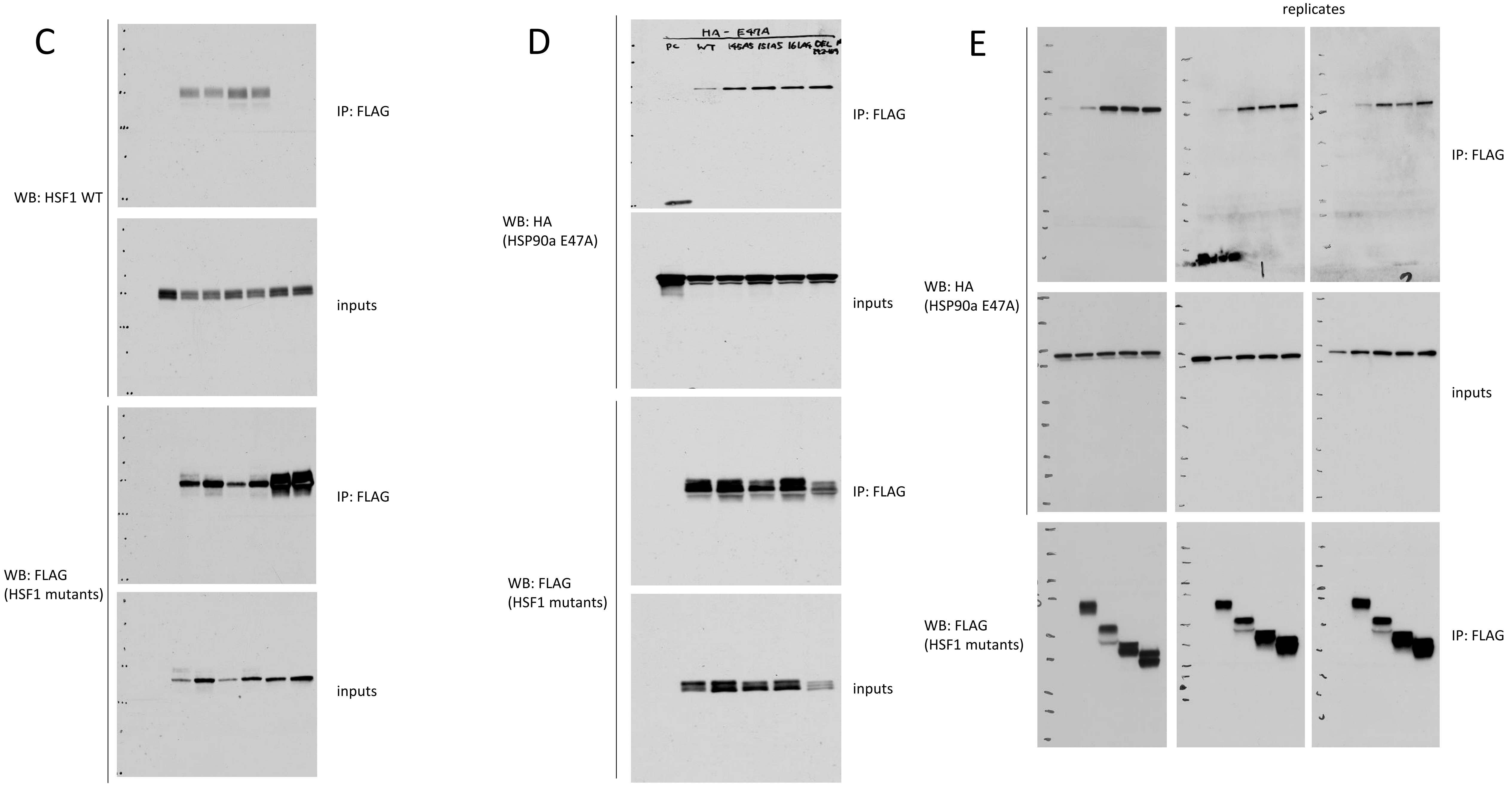

Supplemental Figure 2E cont.

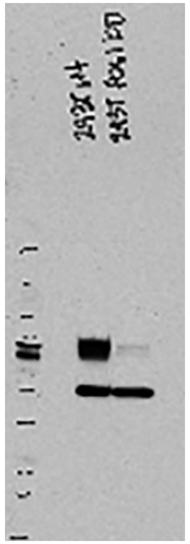

WB: HSF1

WB: α-tubulin

Supplemental Figure 3

C

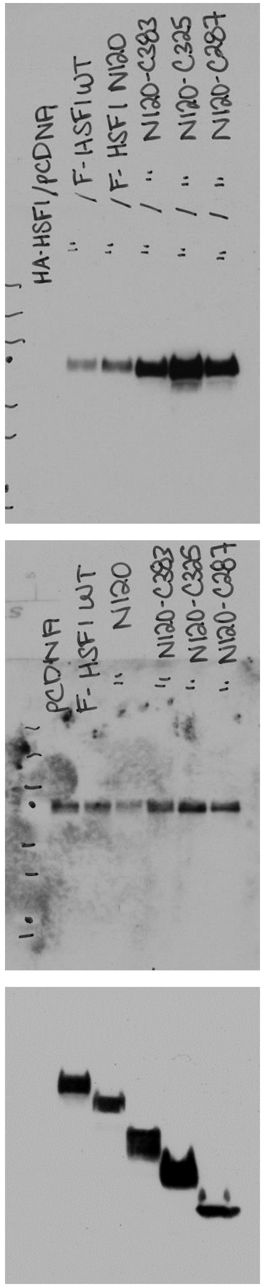

WB: HA (HSF1 WT)

IP: FLAG

inputs

WB: FLAG (HSF1)

IP: FLAG

Supplemental Figure 4

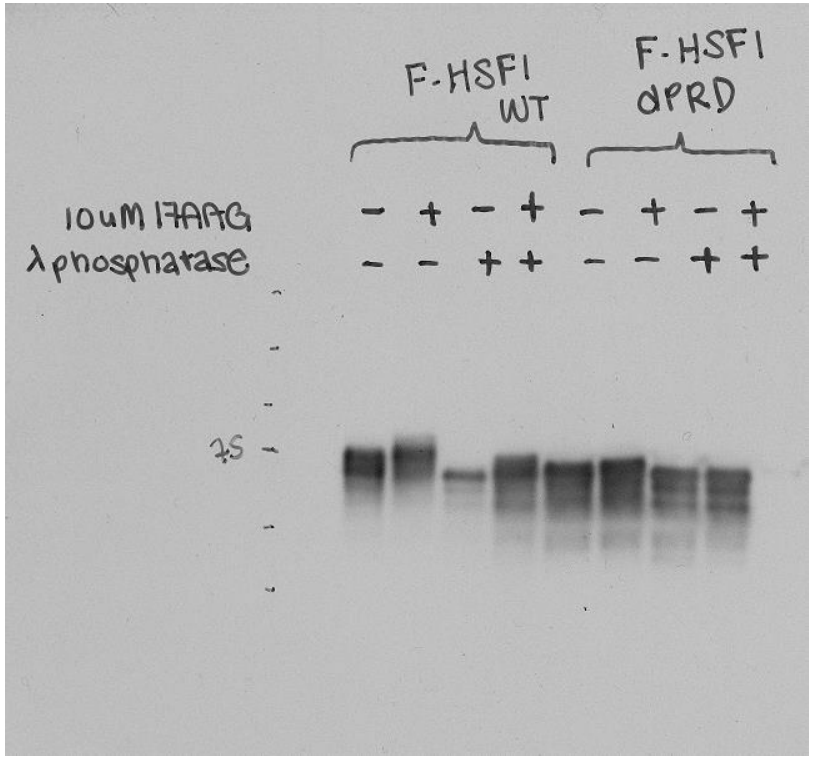

WB: HSF1

IP: FLAG
